# Anxiety and Performance in Sex, Sport, and Stage: Identifying Common Ground

**DOI:** 10.3389/fpsyg.2019.01615

**Published:** 2019-07-16

**Authors:** David L. Rowland, Jacques J. D. M. van Lankveld

**Affiliations:** ^1^Department of Psychology, Valparaiso University, Valparaiso, IN, United States; ^2^Department of Psychology, Open University of the Netherlands, Heerlen, Netherlands

**Keywords:** anxiety, sexual dysfunction, performance, public performance, sports psychology, stage fright, choking

## Abstract

Anxiety has long been associated with diminished performance within a number of domains involving evaluative interpersonal interactions, including Sex, Sport, and Stage. Here, we pose three questions: (1) how do these disparate fields approach and understand anxiety and performance; (2) how does the understanding of the issue within one field offer insight to another field; and (3) how could each field benefit from the ideas and strategies used by the others. We begin with a short review of models of anxiety/arousal and performance and then explore definitions, models, presumed underlying physiological processes, and characterizing and influencing factors within each domain separately in a narrative review. This discussion is followed by a synthesis that identifies elements specific to and common across the various domains, with the latter captured in a model of essential characteristics. Concluding remarks note the potential value of promoting increased cross-disciplinary conversation and research, with each domain likely benefiting from the conceptualizations and expert knowledge of the others.

## Introduction

Anxiety has long been associated with performance problems in a variety of fields (Masters and Johnson, 1970; Kleine, 1990; McCabe, 2005; Kenny, 2011; Oudejans et al., 2013; Biasutti and Concina, 2014) and, not surprisingly, remediation often includes anxiety reduction techniques as a key element for addressing the problem. Anxiety, defined as a negative mood, typically accompanied by bodily symptoms such as increased heart rate, muscle tension, a sense of unease, and apprehension about the future (Barlow, 2002; American Psychiatric Association, 2013), can serve as a strong motivator for future avoidance and/or approach behaviors.

Anxiety is often tied to any type of performance, particularly when an *evaluative* component is present (Hope and Heimberg, 1988; Bögels and Lamers, 2002). Furthermore, evaluation often entails significant *consequences* for an individual, which may further intensify the anxiety (Bancroft, 2009)^1^. For example, partnered sexual activity, particularly at the outset of a relationship, typically includes both evaluative and consequence components, and sexual impairment has long been associated with anxiety (Barlow, 1986; van den Hout and Barlow, 2000; Dèttore et al., 2013; Gerrior et al., 2015). Each individual in the dyad may be concerned about his/her partner’s expectations and perceptions, and each may worry that if performance is not “adequate,” not only will embarrassment and shame follow, but the relationship itself may be in jeopardy.

In sports, players are often under scrutiny not just by teammates but also by fans, and the consequences for failed or diminished athletic performance can be both personal (embarrassment, loss of confidence, etc.) and professional (loss of contracts, income, etc.). Stage performance shares similar characteristics. Whether the situation is musical, theatrical, or simply public speaking, each consists of evaluation by the audience (and critics), coupled with personal and professional consequences (Biasutti and Concina, 2014). Such anxiety undoubtedly occurs in other activities (e.g., academic test taking and work/professional conditions), but situations involving Sex, Sport, and Stage share the fact that they represent *interpersonal* performance domains (unlike test taking or math anxiety). They are known to provoke anxiety which in turn may affect performance and may eventually lead to avoidance. Well-known examples of anxiety affecting performance in Sport include John McEnroe (tennis) and Phil Mickelsen (golf); examples in Stage include Barbara Streisand, Megan Fox, Leonard Cohen, and Jim Carrey (Jacobs, 2013). Examples in Sex are for obvious reasons not known but consider Fellini’s classical movie *Satyricon*, based on Petronius’ book, in which the protagonist finds himself having to perform sexually in front of clamoring throngs at a Roman amphitheater. Not surprisingly, he gets the thumbs down from the audience!

However, while anxieties related to Sex, Sport, and Stage share the common elements of evaluation and consequence, they also differ in substantive ways. For one, the response sets are different: in Sex, autonomic responses related to sexual arousal are most salient; in Sport, visual/spatial motor responses may often dominate; and in Stage, cognitive/memory (including motor memory) processes are engaged, sometimes fully memorized, other times aided with musical scores or written notes. Second, Sport and Stage are carried out in highly competitive environments, from tryouts and auditions—where competition for coveted positions is intense—to public performances in either solo or team formats. Sex is less competitive, usually an intimate performance involving two people.

The nature of the evaluation may also vary. Beyond self-evaluation—generally common to all three—evaluation in Sport and Stage typically involves larger groups. The consequences, too, may be quite different, more personal and intra-relational for sexual failure, more public and professional for stage and sports failures. In all three situations, however, embarrassment and shame are likely to follow failure, and such failure may present an obstacle to attaining an important life goal (sexual partner, job, wealth, respect).

While all three fields—Sex, Sport, and Stage—deal with performance problems related to anxiety, with general reviews written within each field (e.g., Seto, 1992; Craft et al., 2003; Kenny, 2011; Çırakoğlu, 2013; Dèttore et al., 2013), cross-disciplinary theorizing and conversation has been limited^2^. In transcending disciplinary isolation, mutual benefit might be realized by all three fields, with each gaining understanding and insight from the other.

Herein, we explore three questions: (1) how do these disparate fields approach and understand anxiety and performance? (2) how does the understanding of the issue within one field offer insight to another field; and (3) how could each field benefit from the ideas and strategies used by the others? In addition, we pose a fourth question: whether the anxiety-performance dimension in each of these three disparate domains might be subsumed under a single model, such that common language and interpretation might ensure optimal benefit in both conceptualization and practice across fields.

To this end:

1. We first provide a short review of general models of anxiety/arousal and how they affect performance.
2. Then for each domain (Sex, Sport, and Stage), we explore the issues: definitions, prevalence, models, underlying physiological processes, and precipitating and mitigating conditions in a narrative review.
3. And, finally, we integrate the fields by identifying points of intersection and differentiation and ask whether a unifying model might stimulate hypothesis testing and further research across these fields.

We realize that by first discussing the literature separately for each domain (Sex, Sport, and Stage), we at times repeat theoretical positions on anxiety and performance that occur across domains. Yet, this approach will provide a better sense of the scope/magnitude of the dialogue on each of the various factors related to anxiety and performance, as major lines of thinking/research in one discipline often differ substantially from those of another discipline. Furthermore, as we progress from one domain to the next, we increasingly cross-reference concepts in previously discussed domains to highlight parallel developments occurring within each of the fields. Were we to organize discourse around specific topics/variables related to anxiety-performance and discuss them concurrently across domains, we believe that the original/unique perspectives within each domain would be lost. That is, while core conceptualizations may share common elements across domains, the accoutrements and trappings surrounding each construct are frequently very different across Sport, Stage, and Sex. We trust that, in the final synthesis, the parallel concepts across domains will be sufficiently familiar to make the cross-disciplinary connections easy to understand.

### General Models of Anxiety, Arousal, and Disrupted Performance

A dominant model of the relationship between anxiety, arousal, and performance was published as early as 1908 and became known as the Yerkes-Dodson Law (YDL; Yerkes and Dodson, 1908) (see reviews of Broadhurst, 1959; Broadbent, 1965; Teigen, 1994; Landers, 2007). Yerkes and Dodson postulated that, as stimulus strength rises, habit formation improves, but only up to a certain maximum, when it begins to deteriorate as stimulus strength continues to increase, generating an inverted U-shape function. Task difficulty moderates this relation: the optimal anxiety-provoking stimulus strength is higher for easier than difficult tasks. Yerkes and Dodson’s model is one of the few theoretical models in psychology that attained a law-like status. However, it has also encountered criticisms, including its overly general applicability to situations and performance types to which it has been applied (see Neiss, 1988, p. 345; Hanoch and Vitouch, 2004; Corbett, 2015). Other frameworks, including learning theory, cognitive/motivation theory, and stress-coping theories (e.g., Lazarus and Folkman, 1984; Lazarus, 2000) can also be invoked to account for individual variability and changes over time regarding the effect of anxiety on performance.

A more recent type of cognitive theory—and in our view more relevant and explanatory—regarding anxiety’s impact on performance in Sex, Sport, and Stage is the reflective-impulsive model (RIM: Strack and Deutsch, 2004; see also Ouimet, 2017). The RIM belongs to a larger “family” of dual-process models (Evans and Frankish, 2009) comprising two highly different, independent, and component cognitive processes. The phylogenetically older component is the impulsive system, an intuitive modus operandi with operands formed by accumulated experience. The impulsive system is permanently active and operates automatically as it processes incoming information from the entire perceptual field, requiring minimal cognitive resources (Evans, 2003). Most of its operations occur outside awareness, although some may become part of the conscious experience. The other component is the reflective system, the system involved in abstract, conditional, and hypothetical reasoning. As this system requires holding several bits of information in working memory, it can handle only small amounts of information and is highly dependent on the availability of processing capacity in working memory (Baddeley and Hitch, 1975; Evans, 2003). A common example of dual processing is expert car driving. A driver effortlessly performs all necessary adjustments while processing information from both the traffic situation and the car systems and yet may be simultaneously deeply immersed in a conversation with a passenger. However, when traffic information signals danger, the driver interrupts the conversation to refocus attentional resources to respond to the traffic situation. The reflective system interrupts or overrides reflexive processing and thus can exert inhibitory control (Evans and Stanovich, 2013; but see Newell and Shanks, 2014, for a critical review). If for some reason processing capacity is reduced, the omnipresent reflexive system will assume priority in the control of behavioral output (Hofmann et al., 2009).

**Figure 1:**
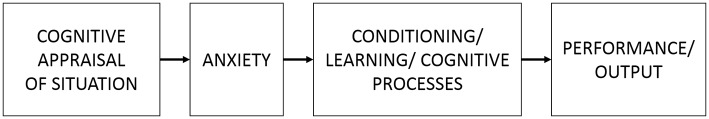
Scope of discussion addressing anxiety and performance.

Although we have thus far referred primarily to “anxiety” and “performance,” different domains approach the issue in slightly different ways. For example, the incapacity to perform under pressure is described as “choking” during sporting events, with primary focus on performance rather than anxiety. In stage performance, the literature focuses on “stage fright” and its impact during stage performance. In sexual response, emphasis is on “performance demand” and its relationship to sexual dysfunction. Although terminologies vary, and the reference point in the anxiety-performance relationship may shift, in each case, poor performance is typically associated with excessive anxiety related to fear of failure in an evaluative context. Vulnerability to the effects of anxiety is often framed using a variety of learning/cognitive, situational, and personality factors (see Figure 1 for the relevant constructs and the relationships that form the basis of this paper’s discussion).

## Method

### Selection of Databases

For this integrative review, the following databases were accessed: SPORTDiscus with Full Text, International Bibliography of Theatre and Dance, Music Periodicals Database, RILM Abstract of Music Literature with Full Text, PsycARTICLES, PsycINFO, and MEDLINE/PubMed.

### Search Strategy

Depending on the database, all possible combinations (using the Boolean connector “AND”) were made across the following categories of keywords. Category 1: Sports or Athletics or Competition, Public Speaking, Theatre or Theater or Drama, Sexual or Sex; Category 2: Performance; Category 3 (depending on Category 1 entry): Anxiety, Fear, Freezing, Stage Fright, Choking, Dysfunction, Forgetting, Cognitive, Affective, Behavior. We also set the following parameters: Keywords found in any part of the text; search 1970 or after; English language; Scholarly Articles (with and without peer review).

## Results

### Anxiety and Performance in Sex

#### Definition and Prevalence

The concept of performance anxiety related to sex has long been known but was first “clinicalized” by Masters and Johnson (1970) in their classic work *Human Sexual Inadequacy*. Sexual performance anxiety refers to the fear that an individual will not measure up to some preconceived expectation within the context of sexual interaction. Whether related to concerns about body image, one’s masculinity or femininity, or aspects of sexual response itself, such anxiety can disrupt normal sexual response and result in unsatisfying sex with one’s partner. Unlike the somatic/voluntary responses of Sport and Stage, aspects of the sexual response—erection, lubrication, orgasm, ejaculation—involve the autonomic nervous (internal) system and thus are not strictly under voluntary control. Although an individual may take steps to increase (or decrease) the likelihood of autonomic activation for arousal and orgasm, such responses cannot generally be “controlled” or “willed” as occurs in Sport or Stage.

Although the extent to which sex-related performance anxiety is a concern within the general population is unclear, sexual situations automatically represent demands on sexual performance (Barlow, 1986), suggesting a fairly pervasive phenomenon. Fairly recent data (Laumann et al., 1999; Mitchell et al., 2013) indicate that about 5–25% of men and women report anxiety surrounding sex—perhaps an underestimate. The estimated prevalence for erectile dysfunction (ED), for example, varies widely and differs according to the type of sex, age group, the specific problem (e.g., getting vs. keeping an erection), and relationship duration. What can be stated with reasonable confidence is that sexual performance-related anxiety and subsequent dysfunctional response occur in a significant portion of men and women at some point in their lives (McCabe, 2005; Lewis et al., 2010).

#### The Role of Biological Factors in Sexual Performance Anxiety

A physiological basis for anxiety’s role has been delineated for several sexual dysfunctions. For example, a high level of anxiety (and stress) may interfere with the normal erectile process: anxiety typically prompts elevated sympathetic nervous system response (flight or fight), whereas the process of erection demands a predominantly parasympathetic response. Furthermore, elevated cortisol (a stress hormone) has been associated with diminished erectile response and greater self-reported “worry” (Rowland et al., 1987). As men progress through the sexual response cycle, dominance typically shifts from parasympathetic to sympathetic control necessary for ejaculation. Evidence suggests that some men show a too-rapid shift from parasympathetic to sympathetic dominance, resulting in (a premature) ejaculation before the man feels ready (Rowland, 2011; Rowland and Crawford, 2011).

In women, the relationship between anxiety and sexual response is less clear. Inducing an anxiety state may in some instances increase sexual arousal (Meston and Bradford, 2007; Meana, 2012), but most often, anxiety interferes with all phases of sexual response—desire, arousal (including lubrication), and orgasm (McCabe, 2005)—perhaps in a manner consistent with the YDL. Unlike in men, in women, the understanding of the effect of anxiety on autonomically controlled aspects of sexual response is not well delineated. However, to the extent that anxiety may serve as a distractor from erotic cues, it may negatively affect all aspects of sexual response in both men and women (Geer and Fuhr, 1976; Farkas et al., 1979; van Lankveld and van den Hout, 2004; Salemink and van Lankveld, 2006).

#### Psychological Models of Sexual Performance Anxiety and Course of Development

Several models, two general and one focused on the role of performance anxiety, have attempted to understand sexual response and dysfunction.

*Inhibition-excitation models* focus on the opposing effects of excitatory and inhibitory factors rather than on performance demand *per se* (Bancroft and Janssen, 2000; Perelman, 2009). In these models, excitatory factors may be individual, relational, and contextual—they include both neurobiological and psycho-socio-cultural factors. For example, the desire and attraction to one’s partner, and the value of sexual intimacy and a satisfying relationship represent important excitatory elements. Inhibitory factors—ones that interfere with the sexual response—also involve individual and bio-psycho-socio-cultural components and may include a neurobiological predisposition for anxiety, relationship conflict that suspends sexual advances, or cognition/assessment of risk factors resulting from infectious disease, inappropriate (and sometimes illegal) objects of desire, and so on. In such models, performance anxiety assumes a role as one of any number of inhibiting factors on sexual response.

More directly focused on anxiety performance related to male erectile response and to a lesser extent female arousal inhibition, Barlow (1986) proposed a *cognitive-affective model* that distinguished sexually functional men from dysfunctional men through feedback loops. According to this widely referenced model, sexually functional men progress through stages that lead to stepwise increases in autonomic arousal, subsequent functional performance, and future approach toward similar situations. In contrast, dysfunctional men progress through similar stages, yet due to low expectancies, self-efficacy, perception of control, and attention on consequences of failure rather than on erotic cues, these variant stages lead to autonomic arousal/anxiety, dysfunctional performance, and avoidance in future situations. This descriptive model generated a quest to verify differences between dysfunctional and functional men along a number of dimensions; however, the model did not address either how the dysfunctional response developed in the first place, or why some men develop performance anxiety and others do not.

Although the precise effect of psychogenic factors on sexual response is difficult to disentangle, men and women nevertheless often worry, show concern, or are otherwise anxious about the “adequacy” of their sexual response, and as this worry increases, it has, ironically, increasing probability of disrupting it (Bancroft, 2009). And, if one failure follows another, the level of anticipatory performance anxiety is likely to increase, which then further interferes with sexual response. In a typical fashion, the individual attempts to “pursue” the response (e.g., erection in men, orgasm in women) rather than to allow it to “ensue” in response to sexual stimulation. Such compounding failures impart both emotional and cognitive effects—the individual may become obsessed with negative thoughts related to failure, embarrassment, and shame (Bruce and Barlow, 1990; Rowland et al., 1995, 1996; Nobre and Pinto-Gouveia, 2008), augmenting his/her frustration, sense of low self-efficacy, and anxiety—all of which further decrease the likelihood of an adequate sexual response. Indeed, an individual’s actions may become directed more toward reducing the anxiety (e.g., attempting successful penetration before fully becoming erect) than toward the positive feelings that usually accompany sexual intimacy.

#### Characteristics and Influencers of Anxiety Surrounding Sexual Performance

For both men and women, the relationships between anxiety and performance are neither simple nor straightforward. For example, men and women having no sexual problems sometimes experience enhanced arousal under conditions of anxiety, but for those already having sexual difficulties, anxiety tends to compound the problem—they exhibit even stronger susceptibility to the effects of performance demand (Barlow et al., 1983; but also see Beck and Barlow, 1986). Some evidence for a curvilinear relationship between sympathetic activation and sexual arousal, similar to the YDL, has been indicated (Lorenz et al., 2012). Not surprisingly, given the anxiety surrounding performance, men and women with sexual problems report higher levels of negative affect in anticipation of and in response to sexual experiences than functional men (Rowland et al., 1996; Meana, 2012). A number of factors are likely to cause, result from, or exacerbate such anxiety.

##### Self-Focus

Both men and women with performance anxiety tend to focus heavily on themselves rather than on the erotic cues provided by the partner. This self-focus includes the monitoring of one’s own physical responsiveness [e.g., for men, the extent of their erection; for women, how close they are (or are not) to orgasm], a process referred to as “*spectatoring*” or “*hypervigilance*” (Masters and Johnson, 1970). For most individuals, close self-monitoring has negative results: arousal due to erotic cues is either lost or never achieved because the focus on one’s own body—whether related to body image, genital pain, or sexual response itself—precludes attention to partner-generated erotic cues (van Lankveld et al., 2004; Meston, 2006; van Lankveld and Bergh, 2008; Woertman and van den Brink, 2012). A curvilinear relationship, similar to the YDL, has also been suggested between self-focus level and sexual arousal (van Lankveld et al., 2004; Meston, 2006).

##### Distraction

One consequence of self-focus is that it distracts from the erotic cues at hand. This shift in focus counters the typical (and somewhat reflexive) response to those cues, such that levels of psychological/physiological arousal necessary for erection or lubrication are never achieved (Geer and Fuhr, 1976; Farkas et al., 1979; Salemink and van Lankveld, 2006). Although self-focus and distraction often occur concomitantly—resulting in diminished performance—the parameters may not be the same for the two sexes. While sexually dysfunctional men tend to self-focus on their erection, how aroused they are, or how incompetent they are, sexually dysfunctional women tend to self-focus on their body appearance and, surprisingly, non-sexual residuals from the day, although thoughts about incompetency also play a role (Nobre and Pinto-Gouveia, 2008, 2009).

##### Expectancies and Self-Assessment

Men and women with sexual problems tend to underestimate (or at least underreport) their level of subjective arousal and genital response (Heiman and Rowland, 1983; Barlow, 1986). Two cognitive processes might explain this. (1) These individuals may set high (or even unattainable) expectations for themselves, based on what they themselves want, what they believe their partners expect, and what they assume to be a socio-cultural norm; that is, they believe that they can never live up to the expectations they have adopted. (2) These individuals are also more likely to catastrophize about their situation, believing that the situation is far worse than it really is.

##### Diminished Self-Efficacy and Negative Scripts

The above processes often result in a diminished “self-efficacy,” a construct developed by Bandura (1989) that refers to one’s perceived ability to be effective at a given task based on previous experiences (Albarracín et al., 2001). Individuals with high self-efficacy rehearse situations with positive performance strategies and visualize success even when having to overcome significant problems, whereas those with low self-efficacy dwell on the negatives of the situation and envision failed scenarios (Bandura, 1989).

Men and women who have recurring sexual failures begin to view sexual situations differently—no longer as an opportunity for pleasure and intimacy but as a situation that leads to failure, shame, and embarrassment (Rowland et al., 1996; Desrochers et al., 2009). Thus, from a cognitive/affective perspective, these individuals handicap themselves. Their self-narrative (thoughts) during sex becomes negative, with the inevitability of failure as the anticipated outcome of any encounter. Concomitantly, anxiety levels overwhelm any potential for positive affect, thereby engendering counterproductive behaviors—including avoidance of intimacy altogether—that are aimed at reducing the negative affect but which often only sustain or intensify the problem (Fichten et al., 1988; Rowland et al., 2015).

#### Vulnerable Personalities

Psychologists have long sought a link between various personality characteristics and a propensity toward psychogenic sexual problems to better understand why some individuals seem more vulnerable than others. Several personality traits have a fairly straightforward relationship to sexual arousal, pleasure, and dysfunction (Quinta-Gomes and Nobre, 2011). Specific personality characteristics may render the individual more vulnerable to the evaluative components of sexual interaction and raise the person’s anxiety by placing high value/effort on sexual performance, inducing fear of failure and negative consequences. Whether such anxiety is specific to the sexual situation or represents a broader personality tendency, the experience of anxiety surrounding sexual activity may shape sexual attitudes and expectations, which exacerbate existing or developing sexual difficulties.

Although such traits may add to either sexual enjoyment or sexual problems, they represent only one of many factors impinging on sexual response for a given individual with a given partner. That is, personality characteristics interact with various relational and situational factors, resulting in specific vulnerabilities or strengths in situations of sexual intimacy. As such, they probably play a secondary role—perhaps exacerbating or intensifying—in sexual response and enjoyment.

##### Negative Trait Affectivity

Negative trait affectivity is characterized by an undue focus on negative emotions (e.g., anger, anxiety, and guilt), whereas positive trait affectivity is characterized by a focus on positive emotions (happiness and enthusiasm). Negative trait affect is associated with problems surrounding sexual functioning and sexual pain in women (Oliveira and Nobre, 2013) and diminished arousal and erectile functioning in men (Peixoto and Nobre, 2012).

Related to negative trait affect, high levels of *neuroticism*, a personality trait characterized by nervousness, fear, worry, and lower emotional stability, have been associated with male sexual dysfunctions. Neurotic tendencies may prompt negative feelings and thoughts; therefore, when sexual issues arise, a man higher in neuroticism is likely to evaluate himself negatively, favoring *internal attributions* for any sexual failure. In women, lower emotional stability is correlated with increased risk of orgasmic difficulties and sexual pain disorders (van Lankveld et al., 2010).

##### Extraversion-Introversion

Extraversion-introversion (E-I) is a characteristic that denotes the source of the person’s psychological, emotional, and (even) physical energy, thereby playing a possible role in sexual enjoyment and dysfunction (Harris et al., 2008; Quinta-Gomes and Nobre, 2011). Although introversion itself does not lead to sexual problems, an introvert may avoid sex due to its emotionally taxing nature, particularly if the individual has recently entered into a new relationship. Similarly, individuals with *social phobia*—intense anxiety elicited from social situations—may respond particularly negatively to the overwhelming pressures (both cultural and partner-based) to perform adequately during sex (Figueira et al., 2001).

In summary, a variety of situational, cognitive/behavioral, and personality factors have been invoked within the field of sexology to explain the anxiety-performance connection.

### Anxiety and Performance in Sport

#### Definition and Prevalence

“Choking” is the term most often used for any instance of poor performance in a professional Sport. In contrast with “performance anxiety” used in the context of sexual response—which refers to a particular situational pressure to respond adequately—choking refers to the inadequate response itself. Analogous terminology in sexology might be “impaired” or “dysfunctional” response, in Stage, “freezing” or “substandard performance.” The terminology “competitive anxiety” in Sport refers to the elevated stress/anxiety that occurs in situations when the demands of training or competition exceed an athlete’s perceived ability.

Although choking has been described in various ways (see Buszard et al., 2013; Mesagno and Hill, 2013; Mesagno and Beckmann, 2017), an important distinction exists between choking and athletic performance anxiety: performance anxiety, a feeling commonly experienced by performers, may either enhance or disrupt performance (Alter et al., 2010; Dias et al., 2012; Brooks, 2014; Akinola et al., 2016), perhaps as the YDL inverted U-function suggests, analogous to the manner in which pressure/anxiety may enhance sexual response in some individuals but disrupt it in dysfunctional/vulnerable individuals (Jones et al., 1993; Duncan et al., 2016). Thus, choking refers to the substandard performance (judged by either self or audience standards) occurring under pressure conditions (Baumeister and Showers, 1986) that may include rewards/punishments, evaluative audiences, competition, ego threat, and one-chance events. Baumeister (1984) summarizes this pressure as any factor or combination of factors that increases the *importance* of performing well (see also Otten, 2009). Consistent with this approach, Mesagno et al. (2008) defined choking as a “critical deterioration in the execution of habitual processes as a result of an elevation in anxiety levels under perceived pressure, leading to substandard performance.” Beilock (2010) and others qualify the concept, noting that performance quality may either increase (e.g., “peak” performance) or decrease, with the latter not necessarily labeled as choking, as this occurs only when cognitive appraisal of a given situation as stressful is coupled with suboptimal performance (Anderson et al., 2014; see also Neil et al., 2012).

Other authors have emphasized various aspects of choking. For example, “expectation” has been incorporated into some definitions, with “choking as an inability to perform up to previously exhibited standards” (Daniel, 1981); or both expectation and pressure have been included, for example, choking involves perceived pressure, both internal and external, which results in suboptimal performance in relation to the expectations of the performer (Gucciardi et al., 2010). Still others have included the concept of motivation, specifying that choking occurs only if the performer could obviously have done better and had intended to do so (Lazarus, 2000; Beckmann et al., 2013). Finally, while some definitions focus on prior experience or skill, others examine any instances of performance under pressure. Thus, according to several definitions, novices cannot really “choke” under pressure, as some level of expertise is assumed. In fact, some Sport studies have examined the effects of performance anxiety in novices and experts as distinct phenomena.

Choking occurs in many sports, particularly those involving skill (e.g., tennis, basketball, and golf) as opposed to effort (e.g., track and cycling) (Cox, 2012). Data on prevalence have been based mainly on younger players, 20–30 years old, and most research has not differentiated participants’ level of expertise. Equally problematic, no proposed definition of “expertise” has been widely accepted: some studies have examined players in highly ranked divisions to denote expertise, whereas others focused on the duration of experience in the sport, assuming duration translates into greater expertise. Gender of the participant has been understudied as a relevant factor in prevalence rates, and although sex differences relevant to some athletic activities have been documented, findings are not always consistent (Taylor, 1987; Hammermeister and Burton, 2004; Nicholls and Polman, 2007; Modrono and Guillen, 2011; Cohen-Zada et al., 2017).

Because of variation in definition, types of sports activities, levels of expertise among players, and potential sex differences, prevalence estimates vary measurably. Furthermore, as Sport participation is generally voluntary—similar to Stage performance and to some extent more so than Sex—many who do experience performance anxiety may simply opt out of participation with little or no cost to one’s goals in life. Nevertheless, existing estimates based on small and select samples of individuals who elect to play sports typically weigh in around 30–60% (Wang et al., 2004; Mesagno et al., 2008; Otten, 2009).

#### The Role of Biological Factors in Choking

Unlike sexual psychophysiology where relationships among anxiety, sympathetic activation, and diminished sexual response are both plausible and empirically supported, the connection between biological processes, anxiety, and poor sports performance is less clear. That is, a direct effect of anxiety on somatic sensorimotor control (e.g., visual-motor coordination) is less obvious, although recent evidence suggests a clear link between (state) anxiety and disrupted cognitive-motor performance (Lo et al., 2019). Physical factors also play a role. Both poor physical preparation (e.g., toning, exercise) and physical/mental fatigue have been tied to choking (Hill and Shaw, 2013; Silva and Paiva, 2016), with Wang et al. (2004) explaining that increasing physical or mental effort may induce or exacerbate fatigue, although sport participants themselves generally do not assume this association (Hill et al., 2013). Furthermore, as with sexual response, increased levels of the stress hormone cortisol are associated with poorer performance, for example, in golf and tennis serves (Doan et al., 2007; Lautenbach et al., 2014).

One approach hypothesizes a central neurobiological role through hemispheric brain inhibition and activation (Beckmann et al., 2013). When a skill is first being learned, the left hemisphere (associated with language) is more active due to verbal iterations involved with skill acquisition. But once the skill has been well learned and is performed automatically, left hemisphere processing may be inhibited and the right (associated with visual-spatial performance) becomes dominant. Choking, then, results when left hemisphere control is re-activated in response to pressure or anxiety, which then disrupts the automatic execution of a skill controlled by the right hemisphere. Indeed, such explanation may have common ground with “self-monitoring” in sexual response, and as discussed later, this distinction between verbal vs. spatial dominance in the acquisition of skills is likely relevant to the distinction between reflective vs. reflexive modes of information processing.

#### Psychological Models of Choking and Course of Development

One common theme among theories explaining choking is that of *change in focus*. According to explicit monitoring theory [also known as self-focused attention (Baumeister and Showers, 1986), conscious processing (Masters, 1992), and skill-focus (Baumeister, 1984)], choking occurs when automatic skills (well-learned, reflexive responses) become consciously regulated and disrupted due to depletion of working memory, a condition typically brought on by anxiety (Moran, 2016). Explicit monitoring of responses is important for the skill acquisition process and facilitates performance in novices as motoric output progresses through cognitive, associative, and finally autonomous phases. Thus, when a skill is first learned, the player (Reeves et al., 2007) uses verbal representations of the movement to create a mental image of the motor activity. The subsequent associative phase involves practice of the physical movement in connection with the verbal representations. The final autonomous phase is achieved when performance of the motor skill occurs with little conscious effort or forethought. When this autonomous (reflexive) process reverts to a conscious (reflective) process, the performer regresses to an earlier phase and performance suffers as working memory is “allocated” to the monitoring of a given task rather than to its execution (Baumeister and Showers, 1986; Beckmann et al., 2013). For example, a tennis player under stress may revert to consciously thinking about how to swing the racket instead of allowing the process to occur automatically, shifting focus from objective to technique (Mesagno et al., 2009). Thus, choking would only occur when response sets have reached skill-based level, as characterized by automatic sensorimotor processing (Wallace et al., 2005).

A second approach, Attentional Control Theory, posits that choking occurs when working memory is overburdened by task-irrelevant information that then shifts focus away from task relevant information (Baumeister and Showers, 1986; Nieuwenhuys et al., 2008). When performing, a player processes a multitude of external (environmental) and internal (thoughts and feelings) stimuli (Hill and Shaw, 2013), with some stimuli improving focus on the immediate task, and others—as seen with impaired sexual response—distracting and shifting attention toward processing information that is less task relevant. This attentional shift may lead to choking in two ways. First, working memory is re-allocated to task irrelevant information, for example, a performer shifts focus away from scoring a goal to note the audience or the importance of the game (Oudejans and Pijpers, 2009; Hill and Shaw, 2013; Cocks et al., 2016). Working memory thus becomes overburdened, and choking becomes more likely (Beilock, 2010; Ducrocq et al., 2017). Second, when attention shifts to non-task variables such as monetary incentives, audience presence, or evaluative circumstances, pressure to perform increases and may induce anxiety (Janelle et al., 1999; Hill et al., 2010a, 2013; DeCaro et al., 2011). This attentional shift often leaves players with a sense of lost control, partly validated by performers’ reports that their emotional state affected their performance (Gucciardi et al., 2010). The sense of lost control and anxiety appear to be reciprocating: lack of perceived control (or self-efficacy) or the perceived lack of ability to deal with stress and achieve goals under stress, often occurs prior to anxiety, but it often occurs in response to stress/anxiety as well (Skinner, 1996).

A third approach to choking, self-presentation theory, emphasizes anxiety resulting from the evaluated component of performance which threatens the player’s self-image or self-presentation (Schlenker, 1980; Mesagno et al., 2012). For example, audiences create pressure, with size, status, and salience correlated with choking probability (Baumeister and Showers, 1986). While the audience may include both supportive and antagonistic spectators, coaches, recruiters, and teammates (Wallace et al., 2005), their mere presence creates *expectations* by performers (realistic or perceived) based on prior successes (Jordet, 2009a), overall performance importance (Hill et al., 2010b), and pressure from others to succeed (Wallace et al., 2005). The pressure to fulfill these expectations and the resulting self-focus and anxiety are common precursors to choking (Wallace et al., 2005; Jordet, 2009a,b; Hill et al., 2010a, 2013; Hill and Shaw, 2013), often more so than monetary incentives (Liebling and Shaver, 1973; Hope and Heimberg, 1988; Mesagno et al., 2011). As with arousal and anxiety, expectation likely functions as an inverted U-shaped curve, where too little or too much results in suboptimal performance, consistent with the YDL model.

Self-presentation theory may also explain “self-handicapping,” that is, “any action or choice of performance setting that enhances the opportunity to externalize (or excuse) failure and to internalize (accept credit for) success” (Gardner et al., 2015; but also see Berglas and Jones, 1978), an attribution process that also plays a significant role in sexual anxiety and performance in men and women (Rowland et al., 2016, 2018). Players’ anxiety will often lead to self-handicapping (e.g., verbalizing lack of practice, physical/mental ailment, or ill-preparedness) as a means to protect their self-image (Coudevylle et al., 2008).

In summary, each theory provides insight into pathways of increased anxiety to impaired athletic performance. All three have garnered support, suggesting that each explains an aspect of the emotional, cognitive, and/or behavioral components of choking (Reeves et al., 2007; Wilson et al., 2007a,b; Gucciardi et al., 2010; Hill et al., 2010b; Mesagno et al., 2011; Hill and Shaw, 2013; Englert and Oudejans, 2014; Vealey et al., 2014).

#### Characteristics and Influencers of Anxiety Surrounding Sport Performance

Many of the characteristics of individuals suffering performance anxiety related to Sport/choking are similar to those identified in the research literature regarding Sex.

##### Expectations

Personal meaning carries significant weight for athletes. This meaning may take the form of expectations of self and others, personal goals, importance of the event to the player, and self-identity (Gucciardi et al., 2010; Hill and Shaw, 2013). Similar to high expectations, players who set specific personal goals such as improving on a past performance, achieving a certain score or time, or performing well under pressure, impose greater pressure on themselves. As noted earlier, other factors that affect anxiety and choking include the perceived importance of the event (Hill and Shaw, 2013) and the self-identity status of the athlete: an individual identifying him/herself, say, as a golfer will feel greater pressure to succeed (or as importantly, avoid failure) in order to validate his or her self-identity (Wang et al., 2004; Gucciardi et al., 2010).

##### Vulnerable Personalities

Performers high in *self-consciousness* are more aware of and concerned with others’ impressions of them. Although this need to appear competent can lead to greater anxiety and pressure (Mesagno et al., 2008, 2009, 2012; Hill et al., 2010a), low self-consciousness has also been associated with choking. A *lack* of self-awareness while under pressure may lead some players to become aware of others’ impressions for the first time during a particular event and thus they choke (Baumeister, 1984; Baumeister and Showers, 1986). Related to self-consciousness, *self-esteem* is also involved: Those having high self-esteem experience greater pressure and more self-conscious and strive harder to meet high performance expectancies, and, as a result, may end up choking (Jordet, 2009a,b).

*Perfectionism* represents another risk factor, as perfectionists often attempt to achieve unrealistic goals (Gucciardi et al., 2010; Hill and Shaw, 2013), a situation comparable to unrealistic expectations often occurring during sexual encounters. Yet, the player’s *interpretation* of perfectionism may also be relevant. In one retrospective analysis, those who choked were self-critical when their perfectionist goals were not achieved, whereas those who did not, view their perfectionism as beneficial to their performance, citing it as a means to increase effort and benefit from mistakes (Hill et al., 2010a). Perfectionism may also result in a greater level of “reinvestment,” a strategy that increases the likelihood of conscious monitoring of thoughts and movements and consequently shifts attention away from task-relevant automatic processing information (Masters, 1992).

As with sexual performance issues, evaluation-based *anxiety* is a common condition among chokers and appears to disrupt the cerebral dynamics important to precision cognitive-motor performance (Baumeister and Showers, 1986; Hanton et al., 2002; Mesagno et al., 2009; Lo et al., 2019). Furthermore, players may interpret physiological reactions (racing heart beat) as signs of anxiety (Gucciardi et al., 2010). As anxiety increases—as delineated in the attentional control theory of choking—players designate more cognitive resources to coping with the anxiety as they attempt to perform, including increased self-monitoring (similar to hypervigilance in sexual anxiety) (Gropel, 2016; Masaki et al., 2017). One interpretation suggests that those who lack the mental discipline to deal with the distraction of anxiety show decrements in performance (Hill et al., 2010b). Another argues that anxiety may actually result in greater attention to relevant stimuli but that the high anxiety causes the players to move less efficiently (Nieuwenhuys et al., 2008).

The relevance of the *type of anxiety*—state vs. trait—has not led to consensus, the former referring to fear or worry stemming from a specific context or situation, the latter to a personality factor predisposing an individual to experience ongoing anxiety over time and different contexts. State anxiety presumably affects “processing efficiency” more than trait anxiety (Oudejans et al., 2013), yet those at risk for choking also tend to be high in trait anxiety (Mesagno et al., 2008). Whether the fear of negative evaluation is tied to a specific task (state) or stable dispositional factors (trait) may not be a strong differentiating factor; the two might operate incrementally together, with high trait anxious persons reaching a tipping point toward impaired performance under smaller increments of state anxiety than low trait anxious persons (see also Perelman, 2009, with respect to sexual impairment).

Similar to men and women with low self-efficacy in sexual situations, players with *low self-confidence* (often related to self-efficacy) are more likely to experience anxiety; in contrast, high self-confidence may contribute to superb performance (Hill et al., 2010b; Hazell et al., 2014; Zagorska and Guszkowska, 2014; Rowland et al., 2015). High self-confidence typically leads to greater perceived control, a significant predictor of good performance (Otten, 2009). Yet, Hill and Shaw (2013) found that both high and low self-confidence were risk factors for choking (indicating a YDL function), suggesting that high confidence could lead to overconfidence and cause players to relax and lessen attentional control over task relevant stimuli (i.e., “Dunning-Kruger effect:” Kruger and Dunning, 1999).

Finally, lack of practice and preparation (including poor physical care) has been cited as reasons for choking (Hazell et al., 2014). Chokers may place more value on other obligations than toning and practicing for an event. Interestingly, those who choke for such reasons are more able to rationally examine their performance afterwards (Hill et al., 2010a), suggesting awareness of the relationship between lack of practice and impaired performance.

In summary, Sport and Sex share many common ideas in the study of anxiety-performance, including biological process, psychological strategies (e.g., attribution, self-focus, distraction/attentional factors, expectation level, self-efficacy), and personality characteristics (e.g., anxiety prone, perfectionism, self-confidence, etc.).

### Anxiety and Performance on Stage

#### Definition and Prevalence

Referred to as stage performance anxiety (also stage fright, jitters, or butterflies), this condition characterizes many persons called upon to perform music, public speaking, and acting. Among these, fear of public speaking (glossophobia) is arguably the most widely experienced. Nearly everyone is called upon to give a presentation in his or her lifetime, whereas participation in music or acting events is often voluntary (Do you suffer from glossophobia?, 2001; Lazarus and Abramovitz, 2004; Gaille, 2013; Statistic Brain Research Institute, 2013). Response sets for these various activities differ, but because of the situational similarities, we discuss them under a single domain so as to identify common elements for any stage performance situation.

Performance anxiety on stage is analogous to Sex and Sport anxiety, in that heightened apprehension and negative emotional states are postulated to interfere with desired outcomes (Matei and Ginsborg, 2017). At the same time, it differs from choking in Sport by emphasizing the process rather than the outcome alone. Klickstein’s (2009) definition of music performance anxiety represents a typical approach, characterizing stage anxiety as stress that interferes with performance. Other definitions are more elaborate, with music anxiety being defined as the “experience of persisting, distressful apprehension, and/or actual impairment of performance skills in a public context, to a degree unwarranted given the individual’s musical aptitude, training, and level of preparation” (Salmon, 1990) or as “the experience of marked and persistent anxious apprehension related to musical performance that has arisen through underlying biological and/or psychological vulnerabilities and/or specific anxiety-conditioning experiences” (Kenny, 2009, 2011). From an acting perspective, stage fright has been characterized as an “unmooring terror” which overwhelms the actor as he/she is about to take the stage, with the actors’ stress level on opening night equal to that of a car accident victim: Stage fright is “a traumatic, insidious attack on the performers’ expressive instrument, their bodies” (Lahr, 2006).

Studies suggest that performance anxiety is pervasive among stage performers, most having experienced it at some point during their career. In music performance, depending on the methodology, estimates indicate 50–70% of professional musicians admit to compromised performances (and larger consequences as well) from anxiety (van Kemenade et al., 1995; Kirchner et al., 2011; Klickstein, 2009; Kenny, 2011; Helding, 2016), with variation dependent on such factors as solo vs. group performance, audience make up (are family members present?), level of performance of fellow performers, and so on. Although prevalence rates for glossophobia are hard to come by—perhaps because the assumption is that, given the right conditions, nearly everyone experiences it—several sources suggest that about 75% of the population has suffered from this condition at one time or another (Statistic Brain Research Institute, 2013).

#### The Role of Biological Factors in Stage Performance Anxiety

Most thinking regarding stage performance anxiety invokes autonomic processes, emotion, and inhibition as factors. For example, sympathetic arousal is responsible for the increased heart rate experienced by musicians suffering from performance anxiety (Finn et al., 2009; Klickstein, 2009; Yoshie et al., 2009). Anxiety increases sympathetic activation and glucocorticoid (stress hormone) release in musicians and those in public speaking situations (Kirschbaum et al., 1992; Fancourt et al., 2015), which in turn may have deleterious effects during performance: it increases fatigue, affects “flow” (a state of focused absorption in an activity: Csikzsentmihaly, 1990), alters the temporal perception critical for the rhythm and pace of musical performance, and disrupts general mental processing (Kirchner et al., 2011; Yoshie et al., 2009). The level of sympathetic activation (e.g., as assessed by heart rate) depends on numerous factors, such as the setting, situation, and performance type, for example, whether public or semi-private (Finn et al., 2009).

Other biological factors such as “physical reactivity” are viewed as important correlates of stage performance anxiety (Fredrikson and Gunnarsson, 1992; Finn et al., 2009). Finn et al. posit those with a “weak” (sic) nervous system can be more (physiologically) sensitive to aversive stimuli than those with a “hardy” (presumably, more resilient) nervous system. As with Sport/choking, stage performance anxiety has been explained in part by the Behavioral Inhibition and Activation System (BIS-BAS) model, although in a different manner^3^. On stage, the Behavior Inhibition System (BIS) is presumed to regulate the amount of reactivity of the nervous system such that when the body perceives signs of adversity, novelty, and non-reward, BIS neural circuits are activated. The BIS is a “watch and wait” system that monitors general levels of perceived threat. BIS dominance has been linked to psychological state anxiety, which suppresses communicative behaviors such as voice inflection, hand and arm gestures, and facial expressions. Thus, psychological and physiological conditions associated with public-speaking and general stage anxiety involve BIS activation, resulting in dampened behavioral reactivity and responses (Finn et al., 2009).

#### Psychological Models of Stage Anxiety and Course of Development

Models of stage performance anxiety have drawn substantially from theories of general anxiety and activation, although different areas (music vs. acting vs. speech presentation) have sometimes emphasized different aspects of these theories. Similar to Sex, Stage performance anxiety is viewed as an interactive process of cognitive, behavioral, and physiological factors, involving, for example, distressing thoughts, autonomic arousal, and impaired behavioral responses (Hoffman and Hanrahan, 2012; Walker and Commander, 2017). Other characteristics include biological vulnerability, high trait anxiety, fear of evaluation, history of “stage nerves,” specific task requirements and/or situations, and distraction.

A somewhat more specific model—though now quite dated—had been proposed for public speaking situations (Behnke and Beatty, 1981)—one derived from Schachter and Singer’s (1962) work—to explain the relationship of psychological and physiological factors to state (vs. trait) anxiety. According to this cognitive-physiological model, a speaker’s response during oration is contingent on two variables: physiological arousal and a cognitive interpretation/labeling of that arousal state. High trait anxiety speakers are likely to interpret their physiological arousal as anxiety or fear, whereas low trait anxiety speakers may perceive the arousal as enthusiasm or excitement (Finn et al., 2009).

##### Person vs. Task/Situation Related Factors

Most research in this field suggests that the causes and/or risk factors for stage performance anxiety may be broadly categorized as either person-related or task/situation-related. Those factors emanating from the individual performer (i.e., *person-related*) include fear of evaluation, lack of preparation, anxiety, and various other personality characteristics.

As with Sport, distinction is made between trait and state anxiety, the former constant across situations and time, the latter varying considerably before, during, and following performances (Finn et al., 2009). Musicians having trait anxiety, thus already having a low threshold for anxious arousal, are more predisposed to a panic (fight-or-flight) behavioral response than more relaxed (low trait anxiety) types (Klickstein, 2009). However, the panic response both results from anxiety and further feeds it, with physiologically aroused states (e.g., heart racing) intensifying the feeling of anxiety. Whereas performers with trait anxiety may develop pervasive nervous habits, which follow them from one performance to the next, those with state anxiety, which results from one’s situation rather than disposition, experience less predictable effects on performance. Because state anxiety, representing an emotional response to a perceived threat, activates the behavioral inhibition system (BIS), the performer’s ability to detect, appraise, and implement appropriate motor responses may be inhibited or even suppressed (Sawyer and Behnke, 2002). Nevertheless, the end effect for state vs. trait anxiety may be quite different: A musician with high trait anxiety walking on stage is more likely to experience panic mode than one with low trait anxiety and low physiological reactivity, even if the latter is experiencing anxiety because of the specific situation (Gosselin et al., 1995; Finn et al., 2009).

Linked to the fight-flight syndrome, *fear of evaluation* is considered another potential cause for performance anxiety among musicians (Helding, 2016). Musical performance involves constant and ongoing evaluation by listeners, so musicians having a high fear of evaluation develop increasing anxiety over the course of their performances, which then can trigger a panic response.

Finally, *poor practice skills* and/or routines are risk factors for anxiety and subsequent diminished performance (Klickstein, 2009; Nicholson et al., 2015). Performers’ habits of practice, more than most other factors, are likely to result in substandard performance resulting from anxiety: “Without the expertise to learn music deeply, on-stage security eludes musicians. Then no matter how simple the material, the performer will not possess the foundations to perform successfully” (Klickstein, 2009). In contrast, when the musician has mastered the performance through strong practice skills, the excitement of being on stage drives a musician to “peaks of artistry.” As with Sport, highly automatized visual-motor behavior based on extensive practice reduces the musician’s vulnerability to shift to de-automatized, controlled performance that is more easily disrupted by apprehensive thoughts, anxiety, and fear.

As with both Sport and Sex, anxiety can be increased by specific conditions (i.e., *task/situation factors*). If a performer does not have adequate preparation time or chooses pieces too challenging^4^, the performer may not be able to master the piece, resulting in low self-confidence and increased stress (Kirchner et al., 2011). Other situational causes include a high level of public scrutiny (when evaluative processes are high, such as performances evaluated during contests and jury judgments), high degree of concern (e.g., consequences of a botched performance), and poor psychological and physical self-care. Even seemingly minor factors may become stressful, for example, a dour stand partner or cantankerous conductor (Klickstein, 2009). Distraction from the audience or co-performers may become catastrophic, although “automatic” performance involving strong focus on the task can help filter such distraction.

##### Short-Term Temporal Onset

Another framework for analyzing the course of stage performance anxiety has been to cluster symptoms according to their temporal sequence, from pre-performance anxiety, to the performance itself, and finally to post-performance factors (Klickstein, 2009). Most pre-performance problems are behavioral, although they may have emotional and cognitive repercussions, may occur hours to weeks before a performance, and include a plethora of possibilities including: procrastination, depression, fatigue, distorted thinking, decreased concentration, interpersonal strife, substance use, and somatic symptoms such as headaches, insomnia, and GI problems.

At performance, symptomatology may occur shortly before and continue through the performance. Both psychological and physical effects may be evident, including somatic symptoms: shaking, cold hands, increased heart rate, profuse sweating, nausea, muscle tension, hyperventilation, dry mouth, and the need to urinate. Mental or emotional symptoms include fear, confusion, memory lapse, distorted thinking, self-doubt, agitation, hypersensitivity, negative self-talk, shame, anger, and panic (Klickstein, 2009).

Post-performance symptoms include an overly-critical evaluation of performance, replaying every slight imperfection, and becoming depressed and frustrated, sometimes turning to substance use to quell emotions and concerns.

Depending on the situation and individual propensities, anxiety onset may differ across types of individuals. Although both “avoiders” and “non-avoiders” feel anxiety on the performance day, “avoiders” are also more likely to experience anxiety mid-performance. Kaplan (1969) details the possible relationship between anxiety and missteps during the actual theatrical stage performance, indicating the following (dramatic!) progression of events: The anxiety begins with manic agitation and moodiness which proceeds to delusional thinking and obsessional fantasies, which then leads to blocking, referring to a complete loss of rehearsed material. The actor then begins to stiffen, shake, and go numb and become uncoordinated, and cognitive processes freeze up causing some actors to dissociate, reporting an out of body experience, thus abandoning the character s/he is playing and losing the illusion of invisibility. Symptoms aside, the important point is that such processes can cause a shift in the cognitive thought process, making the actor more self-aware, “overthinking,” and highly impaired (Lahr, 2006).

#### Characteristics and Influencers of Anxiety Surrounding Stage Performance

Stage performers manifest both unique and shared characteristics relative to Sport and Sex, but within Stage, performers may tend to show individualized or idiosyncratic symptoms.

##### Somatic Symptoms

Reportedly affecting over 50% of performers, somatic symptoms are many, including those listed previously (Witt et al., 2006; Berghs, 2008; Finn et al., 2009; Studer et al., 2011; Hoffman and Hanrahan, 2012). Muscle tonus—either too little or too much—may interfere with performance: increased muscle tension decreases fine motor skills, essential for most stage performance, for example, constricting the body, narrowing the throat, creating tension around the larynx and cheeks, and tightening the abdominal wall (Yoshie et al., 2009). For the vocalist, stage actor, or orator, increased muscle tone makes the voice shrill and poor in vibrato, as if the performer is trying to squeeze the sound out, with fatigue setting in more quickly and making higher notes more difficult. The shoulders become elevated, the neck and back stiffen, often affecting both pitch and tempo of the voice (Berghs, 2008; Hoffman and Hanrahan, 2012). Beyond voiced-based performances, increased muscle tension may disrupt stringed instrument performance by creating stronger keystroke force while playing the keyboards, a significant problem as keystroke force is a fundamental skill for pianists. On the other hand, *decreased* (insufficient) muscle tension may leave the performer less physically coordinated and precise, or even paralyzed by fear. For example, the vocalist’s abdomen will be weak and saggy with shallow breathing, making the voice airy with a slow vibrato and an inability to properly control breathing for long notes (Berghs, 2008). Trembling knees and an expressionless face often characterize decreased muscle tonus (Hoffman and Hanrahan, 2012).

##### Psychological Symptoms

Many performers show psychological and behavioral effects from anxiety, sometimes in conjunction with or more intensely than physical effects (Sadler and Miller, 2010). “Freezing up” and catastrophizing are ongoing problems in all categories of stage performance and include being unable to speak or move during a presentation, forgetting lines in a play, or forgetting the words or notes of a musical piece. Under some circumstances, “de-realization” occurs, such that the performer has a temporary lapse, feeling disconnected from reality and his/her physical body, actually experiencing the self from a distance (Berghs, 2008).

##### Vulnerable Personalities

As with Sport and Sex, certain personality factors have been associated with stage performance anxiety. *Trait anxiety*, identified earlier as a factor, is often correlated with *shyness* and/or introversion, not surprising as this trait is associated with discomfort and lower confidence in social situations. Shy individuals not only experience higher levels of stress but also different kinds of performance stress than outgoing performers (Klickstein, 2009), the former tending to struggle with anxiety and lack of self-confidence in other areas of their lives (Kenny, 2006). Within performance situations, introversion—such as not knowing what to say in social situations—can affect musical performance, acting, and public speaking where some improvisation may be required; a “fixed” performance or presentation, especially one that does not need to be memorized, is both easier and less anxiety-provoking. Related to shyness, stage performance anxiety shares commonalities with *social phobia (social anxiety disorder)*, distinct from performance anxiety in that the condition involves a general fear of social interaction that extends beyond just the fear of public performances. But social phobia and anxiety share similar cognitive distortions (Osborne and Franklin, 2002; Glassman et al., 2014), with performers high in social phobia experiencing higher perceived threat such that the threshold trigger for anxiety may be substantially lower (Kenny, 2009; Goodman and Kaufman, 2014).

As with Sport and Sex, *perfectionism* (and unrealistic expectations) contributes to performance anxiety, as performers with very high standards experience more debilitating anxiety than those with moderate (perhaps more realistic) standards (Mor et al., 1995). Elite performers tend to be acutely aware of the relationship between their perfectionism and anxiety levels, as well as the stress it imposes (Kenny, 2005, 2006). Similarly, *high achievers*, who generally perform well but also impose additional pressures on themselves, tend to be more prone to performance anxiety.

Other personality characteristics have also been associated either positively or negatively with stage anxiety. For example, *neuroticism,* the predisposition for adverse reactions to stress, alienation, and negative emotionality, is a strong predisposing factor for performance anxiety, with one multivariate analysis indicating that negative emotionality predicted over 50% of the variance in individual performance (Goodman and Kaufman, 2014). In contrast, high *self-efficacy*, an overall assessment of the performer’s confidence, as well as an externalized *locus of control* (or attribution), an indicator of performance confidence associated with immediate and specific circumstances, are often associated with lower anxiety and better performance (Goodman and Kaufman, 2014).

In summary, Stage research tends to be highly descriptive in nature. Sport and Stage both place high premium on practice and preparation as ways to mitigate the deleterious effects of anxiety. Stage shows considerable overlap with Sport and Sex in a number of ways: not only are physiological/physical dimensions considered critical to explaining the process but psychological constructs that involve self-focus, expectation, attention/automatic processing, self-efficacy, and attribution are all deemed relevant.

### Performance Anxiety: Synthesis and Integration Across Fields

#### Definition and Prevalence

Each field views anxiety as a pervasive factor affecting performance (see Table 1), with variance in prevalence depending on the specific type of activity. In Sport and Stage, variance occurs across solo vs. group performance. Regarding Sport, solo activities such as golf and tennis stand out (e.g., Cohen-Zada et al., 2017); regarding Stage, solo activities such as speeches and vocal/instrumental performances have higher rates. Solo performance may not only lead to public catastrophes, but the opportunity to compensate through a group effort (e.g., as may be done in theater or a team sport) is absent. On the other hand, in group musical situations, where errors can disrupt the flow of the entire performance, the greater pressure often comes from peers, as the specific perpetrator of the error may be less recognizable to the audience.

**Table 1:** Shared and unshared characteristics of performance anxiety in sex, sport, and stage.^*^

Characteristics	Sex	Sport	Stage
*Motor response set*			
Somatic/fine motor coordination		x	x
Presumed sympathetic interference	x		x
Strong somatic symptomology (GI distress, excessive sweat, etc.)			x
Fatigue	?	x	x
*Proficiency aspects*			
Lack of automatic processing style	x	x	x
Lack of practice and preparation		x	x
Performer identification with “professional” role		x	?
*Cognitive*			
Internal focus/monitoring	x	x	x
Over-expectation/perfectionism	x	x	x
Distorted thinking and self-narrative	x	x	x
Negative effect of distraction	x	x	x
Performance importance/consequence	x	x	x
Self-blame attribution style	x	?	?
*Affective*			
High anxiety	x	x	x
General negative affect	x	x	x
Evaluation apprehension	x	x	x
Self-perpetuating and augmenting anxiety	x	x	x
*Personality factors*			
Trait anxiety	x	?	x
Introversion/social phobia/shyness/high self-consciousness	x	x	x
Low self-confidence/self-efficacy	x	x	x
*Other parameters*			
Females more vulnerable	?		x
Solo performance more anxiety provoking than group/partnered		x	?

* *Question marks indicate that a substantial body of knowledge was not found to support a role for this particular factor but it does not necessarily mean that no role exists for it*.

With regard to Sex, the private nature of sexual intimacy (no verifiable or public record exists), the stigma attached to failed sexual response, and the fact that “anxiety related to sexual performance” may be vaguely defined in the mind of respondents give participants reason and/or motivation to underreport problems in this area. Furthermore, sex-related anxiety often diminishes over time as partner familiarity increases and relationships mature. In contrast, Sport and Stage performance is continually carried out in the presence of new audiences, with new material, and with an ongoing potential for new public embarrassments.

#### The Role of Biological Factors

Both Sex and Stage rely on autonomic (sympathetic) nervous system activation to explain anxiety effects. In Sex, sympathetic arousal presumably interferes with the autonomic responses required for sexual arousal, hence mistimed sympathetic activation disrupts autonomic response. In Stage, sympathetic arousal presumably leads to maladaptive blocking/freezing responses that interfere with cognitive and fine motor performance (e.g., Hunnicutt and Winter, 2011). In contrast, Sport emphasizes the role of physical/mental fatigue and poor physical preparation rather than sympathetic activation, yet all three domains associate high levels of stress hormones (e.g., glucocorticoids) with poorer performance. At the central nervous system level, Sport hypothesizes differences between cerebral control over intentional/deliberate moves during the learning process and (different) cerebral control over automatic responses in experienced players (but also see the next section regarding common threads). In fact, an interesting paradox exists regarding choking: sympathetic activation—presumed to interfere with aspects of Sex and Stage performance—is a requisite condition for intensely physical athletic activities; therefore, anxiety and its underlying sympathetic activation might interfere with Sport performance differently than it does with Sex and Stage performance. Alternatively, distinction may need to be made between sympathetic activation due to physical effort vs. to anxiety. Clearly, all three domains could benefit from further experimental research that actively manipulates and concomitantly measures anxiety, sympathetic activation, motor response, and cognitive processing (e.g., concentration, attention, automatic processing, etc.). Such variables have been studied experimentally in other subfields of psychology and pervade Sport and Sex psychology more than Stage psychology.

#### Models and Course of Development: Reflexive vs. Reflective Cognitive Processing

Due to the high stakes of Sport, theory development has been significant. However, models in all three fields address shifts in attentional focus during performance. In Sport, whether this re-focus is best explained by a shift from goal-directed (automatic) processing to movement-directed (conscious) processing, by distraction rising from extraneous stimuli, or by the worries and anxiety of preserving self-image, is unclear. It might be postulated that any shift in focus away from task relevant stimuli (e.g., goal-directed attention) to task irrelevant information (e.g., movement-directed attention) degrades athletic or stage performance, with cognitive processing shifting from automatic-reflexive mode to conscious-deliberate mode. In Sex, a shift in focus from task relevant (e.g., erotic cues from the partner) to task irrelevant (e.g., self-monitored response) information is also associated with impaired performance. Thus, all three fields share the common process of maladaptive attentional shifts to explain impaired response. Yet Sport and Sex differ in how this comes about: in Sex, sympathetic activation presumably interferes with involuntary/autonomic arousal processes, so the individual self-monitors in order to track whether or not his/her response is successfully progressing toward the desired goal (erection, arousal, etc.). In Sport, response sets are under voluntary control, but due to contextual factors and resulting “worry” about self-presentation, cognitive attention may be distracted away from the stimuli required for automatic processing (sometimes through self-monitoring), thereby generating poorly executed motor responses.

Some types of Stage performance share aspects of Sport and Sex anxiety. For example, oration and acting suffer when attention shifts from the object/goal of the presentation to self-monitoring (a problem identified for performance across all three domains), particularly when memorization is required (“how am I doing,” “what if I forget my lines”). Although specific motor outputs vary over domains (e.g., vocalists, actors, athletes, sexual response), in each, the body may partly serve as the “instrument” of performance. In most areas of Stage, the response set also includes verbal/language memory and output, as is required during presentations, vocal music, and acting. Nevertheless, it appears that the specific response set (whether verbal, somatic, or autonomic) is *less* relevant than its automaticity and reflexivity. That is, shifts in processing of information—whether due to anxiety, emotion, distraction, or other factors—from automatic/reflexive to deliberative/reflective mode appear to be a common element linking Sex, Sport, and Stage anxiety to diminished performance.

Finally, harkening back to the YDL, the inverted U-shaped function may have general applicability to effects of anxiety on performance. But it also appears that a number of other factors related to impaired performance follow a similar function, for example, expectation and confidence, where moderate levels appear to enhance performance but very high or low levels become detrimental.

**Figure 2:**
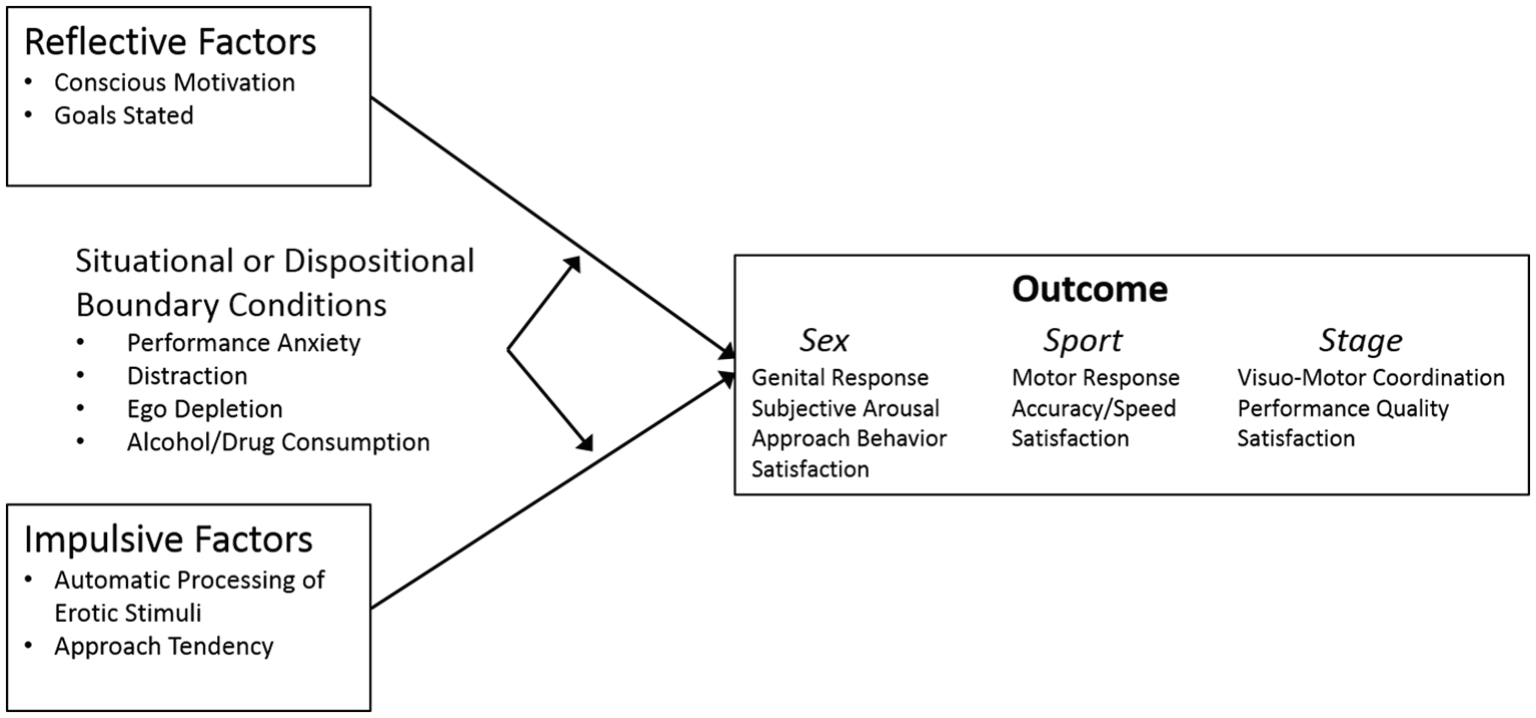
A reflective-impulsive model of the association of performance anxiety and functioning in the domains of sex, sport, and stage performance. Illustration adapted from Hofmann et al., 2009, p.166.

The RIM, as applied to the dynamics of performance anxiety in Sex, Sport, and Stage performance (Figure 2), postulates that both impulsive/reflexive and reflective processing contribute to physiological, emotional, and behavioral outcomes in the domains of interest and that the relative contribution is constrained by situational or dispositional boundary conditions, including performance anxiety, through their effects on information-processing capacity. For each situation, initial engagement requires a learning process involving substantial reflective/verbal operations to generate appropriate responses, but with practice and experience, response sets become automatic, reflexive, and non-reflective. Among other effects, the model predicts that performance anxiety will disrupt automatic, uncompromised functioning in Sex, Sport, and Stage, with this effect mediated in part by a return to (or shift in focus on) deliberative, reflective operations.

#### Characteristics and Influencers of Anxiety and Impaired Performance: General Themes

Besides automatic vs. reflective processing, other elements link Sex, Stage, and Sport anxiety. For example, all three fields recognize the relevance of situation-, task-, and self-related (including personality) factors that may increase (or increase vulnerability to) anxiety and impaired performance (Table 1). These variables do not function in isolation but interact at various levels to increase or decrease anxiety and thus to interfere with or facilitate response sets.

##### Situation and Task Variables

Each domain views evaluation and consequence as relevant variables that can increase performance anxiety and thus impair performance. In Sport, evaluation may emanate from fellow team members, coaches, fans, and even non-fans. In Stage, evaluation emanates from similar sources: fellow players, directors, audience, art critics, and so on. In Sex, evaluation presumably comes from the partner or self-imposed expectations—problems with performance related to erection/arousal, ejaculation, body image, and reaching orgasm all tend to diminish during masturbatory activity in both men and women (e.g., Rowland et al., 2000; Aubin and Heiman, 2004). Thus, across all domains, an increasing sense of evaluation correlates with increased anxiety and consequential effects on attentional focus.

Strongly linked to evaluation is the perceived importance (and thus value) of the situation or event with respect to personal and professional consequences—the greater the perceived consequence, the greater the anxiety. Interestingly, in Sport and Stage, the most serious consequences are related to self-identity, status, and reputation (and thus perhaps future opportunities) rather than immediate financial implications. In Sex, the consequences are more personal, usually related to shame and embarrassment in front of the partner, although—for men vs. women—the presumed norms against which individuals compare themselves are likely different. For example, for women, norms are often tied to attractiveness, appearance, and ability to please the partner; for men, norms may be tied to stamina/endurance, size of erection, and ability to please the partner (Zilbergeld, 1992; Chadwick and van Anders, 2017).

Both Sport and Stage identify other situation- and task-related variables that likely impair or enhance performance. Practice, skill development, and preparation minimize anxiety and impaired performance, whereas inadequate preparation or taking on tasks beyond the player’s competence risk impaired performance. Such variables are explained well by RIM: inadequate preparation and difficult tasks on the one hand, and practice and skill development on the other, represent points along a continuum related to achieving high levels of competency and automatic processing in the expression of response sets. Anything less suggests the need to engage reflective processing—a liability as focus shifts away from automatic processing. Although practice, skill development, and preparation may be less germane to Sex, the analog to “practice” in sexual situations may be “general sexual experience” and/or relationship maturity. Longer, more durable relationships, where commitment and communication are likely more established, may increase “automatized” and reduce self-reflective sexual response, mitigating perceived consequences for sexual failure. However, self-reflection may reappear in individuals experiencing sexual functioning difficulties or starting a new relationship.

Sport and Stage emphasize physical preparation and bodily care, important given that the body may serve as the medium for performance—the athlete’s body must be well toned for precise control, the actor/player’s body in optimal physical health for stage performance, and/or vocalizing. An ill-prepared physical “medium” may increase errors, feed anxiety, and, in an attempt to reduce further errors, shift focus to a more deliberative/reflective mode. The role of physical health in sexual response has received increasing attention over the past two decades though in a different manner. Particularly in men, where cardiovascular disease and diabetes are known risk factors for erectile dysfunction, ongoing cardiovascular and dietary health presumably maintain good vaso-dilative capacity and therefore robust autonomically based genital/erectile response.

##### Person-Related Variables

Distinction is made between person-related factors that arise within specific situations vs. those that are more dispositional and enduring within the individual, a difference not always well demarcated. In either case, person-related factors interact with both the situation and the task to either increase or decrease vulnerability to performance anxiety.

###### Situation-Based Person Characteristics

Individual characteristics may affect a performer’s tendency to become anxious, lose focus, and make errors. For example, in both Sport and Stage, high expectations (and, related to this, perfectionism) may lead performers to set unrealistic goals and then fail to meet them. Perhaps analogous in Sex, novices engaging in their first sexual experience with a partner may “choke” due to overwhelming performance anxiety (Else-Quest, 2014), when their expectations of an ideal performance are very high, perhaps from viewing pornography or hearing exaggerated peer stories.

Low self-efficacy, self-confidence, and self-esteem are characteristics that cluster together, showing substantial overlap. Such factors represent end states (as opposed to traits) that might result from lack of practice and skill development, prior experiences and failures, or taking on overly challenging tasks. Although various domains place greater or lesser emphasis, all three recognize that impaired performance resulting from anxiety can have pernicious self-perpetuating effects: an initial failure leads to distorted and catastrophic cognitions about the probability of ongoing failure, further increasing worry/anxiety and negative self-talk, and setting the stage for future failure (Barlow, 1986; Bancroft, 2009). Thus, the interplay of cognitive, affective, and physiological (autonomic) factors is assumed across all three fields, although the specific ways in which they interact may differ.

Another characteristic mentioned in Sport, though undoubtedly relevant to both Sex and Stage, are the coping skills available to the individual. Although developing appropriate coping skills is often a step toward remediation, many individuals learn and practice homegrown skills on their own. Often these strategies are aimed at controlling or reducing anxiety levels, sometimes by a shift in focus or by reframing, but just as frequently by avoidance, self-medication, or short-term solutions that may interfere with more effective, longer term resolutions.

###### Dispositional Factors

A final cross-disciplinary theme addresses the vulnerable personality, with relevant dispositions clustering around (1) negative affectivity and high (e.g., trait) anxiety; (2) strong self-consciousness and critical self-assessment; and (3) social phobia, shyness, introversion, and fear of evaluation. All three domains—Sex, Sport, and Stage—identify the relevance of such traits to failed performance, although their presumed importance varies across fields.

Men and women high in negative affectivity and trait anxiety have a low threshold for both general and situation-specific anxiety. They are more prone to psychological pressure from any source, including the effects of evaluation and consequence. While anxiety itself may interfere with psychomotor and autonomic response, just as likely, anxiety activates cognitive processes that either deflect focus from automatic information processing or generate counterproductive self-narratives that catastrophize possible negative outcomes.

High levels of self-consciousness and critical self-assessment reflect vulnerability to evaluation and negative feedback. These dispositions likely cluster with close self-monitoring, with judging one’s response against perceived (sometimes unreasonable) expectations, and with experiencing low self-efficacy and self-blame for failure. Such behaviors and mental frameworks, again, are likely to distract the performer from the task at hand.

Finally, introversion, shyness, and social phobia are posited to play roles in both Sex and Stage performance, yet such personal attributes have not assumed a level of significance in Sport. In Stage, such traits place an individual at a clear disadvantage, given the very public nature of most performances. In Sex, the role is less clear and not consistently supported, although such traits may inhibit men and women not only from pursuing broader sexual repertoires (often a strategy to counter sexual impairment) but also from seeking sexual activity in general and/or relationship formation.

In all cases, the fear and experience of failure undoubtedly interact with various personal dispositions and traits, which may in turn (1) result in resilience and coping or, alternatively, (2) perpetuate and intensify the problem. To the extent that specific performance experiences combine with certain personality characteristics (negative trait affect, neuroticism, etc.) to increase an individual’s level of anxiety, shift focus, and affect cognitive narrations, they may lead to or intensify impairment of performance.

## Conclusions and Future Direction

We begin first by acknowledging, as a limitation of the present analysis, that our conclusions are based on a narrative review, which entails the inherent risk of bias of methodology, including—despite our broad literature search—incomplete retrieval of all relevant sources and selective reporting. We also realize that different disciplines may sometimes be tapping into the same (or similar) constructs, although given varying terminology, situations, and assumptions, the “connecting dots” may not always be apparent from specific disciplinary perspectives. And finally, we recognize that although we have treated the issues as somewhat discrete entities (e.g., biological, etiological, personality, situational); in reality, it is impossible to separate such issues into “neat, independent” boxes that singularly affect the relationship between anxiety and performance. A more accurate understanding of the impact of specific factors on the anxiety-performance connection would occur within a dynamic model where factor weights and reciprocations could be incorporated. Despite these limitations, our integrative review has uncovered many points of intersection among the Sex, Sport, and Stage domains.

Although Sex, Sport, and Stage utilize different motor response sets, each entails significant evaluative and consequential components. Context for each of the fields is also different, Sport and Stage often being highly and publicly competitive, Sex generally less so. Nevertheless, the conceptual understanding of the issues, descriptions of mitigating and moderating factors, and approaches toward their understanding share many common elements. Perhaps most striking is the emphasis on the development of an automatic style of information processing and on how anxiety, cognitive distortion, loss of attentional focus, and negative framing interfere with performance by shifting focus to a more deliberative/reflective style of information processing. Each domain further recognizes the importance of situational, task-related, and personal factors in both inhibiting and enhancing performance.

On the other hand, each area is differentiated by its models, assumptions, and specific tactics to achieve cognitive restructuring and automatic processing. Given the high stakes, Sport appears to have taken the lead in some respects, with both quantity and quality of studies that attempt to identify cause-effect relationships. Nevertheless, considerable conceptualization and research have transpired in Stage as well, particularly within music as compared to acting/public speaking. Sex appears to lag behind in the amount of recent/new research on the topic of performance anxiety, perhaps the result of new pharmaceuticals that may indirectly reduce anxiety by increasing sexual performance. Nevertheless, all three domains have much to gain through cross-conversation and fertilization. An agenda for the future might include:

1. Encouraging greater interdisciplinary discussion across domains. The emerging field of Performance Psychology^5^ may serve as a catalyst for such conversation.
2. Comparison of models across domains and determining, for example, whether constructs within each domain might be subsumed under an omnibus model such as the RIM. Kenny (2011), for example, has strongly argued that music performance anxiety is unique and needs to be differentiated from other performance areas, suggesting that a single model cannot be applied across these domains.
3. Identifying parameters of the RIM model across domains that are either common or distinctive. Although this analysis takes an initial step, and support for this model is emerging from empirical research into both male and female sexual functioning (van Lankveld et al., 2015, 2017, 2018), greater model development and elaboration are warranted. Specifically, in Sport and Stage, the potential contributions of RIM-model factors, such as attentional focus and/or information-processing capacity and its reduction by distraction, might provide new insights into anxiety-related performance difficulties in these domains. The idea of “flow” (focused absorption in an activity—perhaps as is seen in the jazz genre) in Stage might share common elements with the use of “mindfulness” during sexual activity for men and women experiencing difficulty, although stark differences in response sets and language/terminology prevent one field of research from easily identifying its counterpart in another field.
4. Carrying out research agendas that test various aspects of the RIM model to specify those most relevant to each domain, for example, examining differences in anxiety between group and singular efforts, low skill and high skill activities, experienced and novice performers, and young and aging performers, and so on. In Stage (music), for example, solo performances appear to generate the greatest levels of anxiety (Spahn et al., 2016), and similar patterns appear to characterize solo sport activities such as golf and tennis (Cohen-Zada et al., 2017).
5. Exploring sex/gender differences—and their potential etiologies—across Stage and Sport. Many differences have been delineated in Sex. In Sport, men show greater vulnerability to choking, at least in one-on-one sports such as tennis (e.g., Cohen-Zada et al., 2017), but findings are inconsistent [e.g., (Taylor, 1987; Hammermeister and Burton, 2004; Nicholls and Polman, 2007; Modrono and Guillen, 2011)], and so, the topic needs further clarification. In Stage, sex/gender differences suggest a pattern whereby women experience greater performance anxiety than men, presumably due to their tendency toward higher trait anxiety and their greater emotional investment in the activity (Hunnicutt and Winter, 2011). The reason for differences in gender/sex effects across domains needs both cataloging and explanation.
6. Examining effective coping and remediation techniques within each domain (e.g., Biasutti and Concina, 2014). Sport and Stage might benefit from sexological approaches that have, for decades, used mindfulness, cognitive reframing, relaxation, and other counseling techniques to motivate and effect significant changes in personal dispositions and frameworks. Indeed, such techniques appear to be gaining traction in both sports and musical circles (Su et al., 2010; Hunnicutt and Winter, 2011; Vaag et al., 2016; Perry et al., 2017). At the same time, Stage and Sex might benefit from the modeling and mental/physical preparation techniques offered by Sport. And Sport and Sex might profit from adopting Stage strategies that help solo performers succeed under enormous pressure and duress.

Perhaps a prime outcome from such a research agenda is to assess whether a “theory of everything” might apply to the study of anxiety and performance, such that a single model might explain anxiety’s effects over disparate areas of performance, ones that involve different response sets, differing competitive environments, and different personal consequences. A second outcome is to identify where details of the model might differ across domains, both in definition and magnitude. For example, in a standard regression or structural equation model (SEM), where anxiety is considered a major predictor variable and performance a major outcome variable, the role (direction and magnitude) of covarying contextual, mediating, and mitigating factors (e.g., as intensity, task difficulty, skill level, dominant cognitive processing style, etc.) needs illumination. In conclusion, we see ample opportunity for all three fields to benefit from cross-disciplinary communication regarding ideas/modeling, various research methodologies and strategies, and new research findings on parallel topics.

## Author Contributions

DR and JL both contributed to the conceptualization, research, writing, editing, and finalization of this article.

### Conflict of Interest Statement

The authors declare that the research was conducted in the absence of any commercial or financial relationships that could be construed as a potential conflict of interest.

## Footnotes

^1^Our focus is on anxiety related to performance, competition, and winning, and not anxiety associated with organizational or personal issues.

^2^For example, a theoretical paper on the shared characteristics of sexual dysfunction and anxiety disorders by van den Hout and Barlow (2000) has attempted to overcome traditional barriers.

^3^For a review of the most recent version of the model, re-named the Reinforcement Sensitivity Theory (see Corr, 2004; Corr and McNaughton, 2008).

^4^This may be analogous to the person who is out on a “dream” date with a highly attractive (and thus highly “valued”) individual and who, at the first attempt at sex with this person, is unable to function sexually.

^5^The American Psychological Association Division 47 launched a journal dedicated to Sport, Exercise and Performance Psychology in 2011 which brings together the study of psychomotor-based performance in a variety of fields, including sports and the arts. See http://www.apa.org/pubs/journals/spy/.
